# Comparison of the Effect of Phenobarbital versus Sodium Valproate in Management of Children with Status Epilepticus

**Published:** 2018

**Authors:** Ali KHAJEH, Fariba YAGHOUBINIA, Saeedeh YAGHOUBI, Afshin FAYYAZI, Ghasem MIRI ALIABAD

**Affiliations:** 1 1.Pediatric Neurology Department, Children and Adolescents Health Research Center, Zahedan University of Medical Science Zahedan, Iran; 2 Nursing Department, Community Nursing Research Center, Zahedan University of Medical Sciences, Zahedan, Iran.; 3 Pediatric Department, School of Medicine, Zahedan University of Medical Sciences, Zahedan, Iran; 4 Pediatric Neurology Department, Hamadan University of Medical Sciences, Hamadan, Iran; 5 Pediatric Hematology-Oncology Department, Children and Adolescents Health Research Center, Zahedan, Iran

**Keywords:** Seizure, Sodium valproate, Phenobarbital, Children, Status epilepticus

## Abstract

**Objectives:**

Acute prolonged seizure is the most common neurological emergency in children. This research was conducted to compare the effect of intravenous phenobarbital and Sodium valproate in control of seizure in children with status epilepticus, referred to emergency ward from Mar to Nov 2013.

**Materials & Methods:**

In this randomized clinical trial, registered with the code number IRCT2015051722300N1, 80 children aged 6 months to 10 years with prolonged seizure and with no response to one dose of diazepam (0.2 mg/kg) administered through IV injection during the five min were selected. Children were randomly divided into two groups, intervention, and control through permutation blocks. In intervention group, intravenous Sodium valproate (20 mg/kg) and in control group, intravenous phenobarbital (20 mg/kg) were prescribed. Data such as age, gender, history of previous seizure, seizure type, and recovery time after receiving drug was recorded in the form. Data analysis was done through descriptive statistics, Chi-square and Independent *t*- test.

**Results:**

Two groups were the same in terms of age and gender and had no statistically significant difference, but they were different in terms of seizure type. In valproate group, 18 patients (45%) and in phenobarbital group, 32 patients (80%) had positive response to the treatment and the chi-square test showed the significant difference.

**Conclusion:**

With regards to this point that in phenobarbital group, patients had more rapid response to drug in comparison with patients in Sodium valproate group, phenobarbital is a suitable and effective drug for controlling of seizure in children.

## Introduction

Seizure is defined as paroxysmal and transient changes in motor, behavioral or autonomic activity followed by disturbance in brain electrical activity (1). Seizure is one of the causes of hospitalization in children and acute prolonged seizure is the most common emergency in Pediatric Neurology Emergency (2). In a six-month random sampling of all of the hospitalized patients in Pediatric Emergency Ward in Amin and Al-Zahra hospitals in Isfahan, central Iran, 60% of hospitalized children had seizure (3). Prolonged seizure is associated with high mortality, especially when it leads to complications such as acidemia, hypoglycemia or hypotension. Timely and effective treatment can improve the prognosis of disease and decrease some complications such as metabolic acidosis and aspiration pneumonia (4).

If seizures last longer than 5 min and the patient does not wake up between them, it is a medical emergency. Status epilepticus is a medical emergency that should be anticipated in any patient who presents with an acute seizure. It is defined as continuous seizure activity lasting more than 30 min or two or more seizures without full recovery of consciousness between any of them lasting for more than 5 minutes. In the past, the cutoff time was 30 min, but this has been reduced to emphasize the risks involved with the longer durations. Moreover, febrile status epilepticus is a febrile seizure lasting longer than 30 min (1).

Since the prevalence of seizure disorders in children is higher than in adults, the using of anti-seizure drugs is high in this age group (5). An ideal anti-seizure drug should have suitable performance, reaches the brain quickly and have minimal side effects. Routinely, benzodiazepines are the first-line treatment of seizures in the emergency (6). 

When the benzodiazepines are not successful in treating seizure, the long-acting anti-seizure drugs should be used (7). In developing countries, phenobarbital and phenytoin are the most common second-line anti-epileptic drugs (8). Phenobarbital was the first antiepileptic drug introduced in 1912. Nowadays, phenobarbital is commonly used for seizure disorders. Although this drug is less effective than phenytoin or carbamazepine, but it is used for treatment of generalized tonic-clonic and partial epilepsies in all age groups (9). Good efficacy, low toxicity and low cost of the drug have suggested it as an important drug for such applications. 

There is evidence that phenobarbital has the most powerful effect in damaging to the behavioral and cognitive activities. The administration of phenobarbital during growth may lead to cognitive impairments such as damage to memory and learning abilities.

Respiratory depression and hypotension are the side effects of rapid phenobarbital infusion, but the most important long-term side effect that limits its use is the impact on children's behavior and learning (10). Sodium valproate as a broad-spectrum anticonvulsant is one of the classic anticonvulsants and is available in our country. This drug is effective in all types of seizures such as absence, tonic-clonic and myoclonic. Besides, it is effective on some kinds of partial epilepsy.

The effect of Sodium valproate and phenytoin in patients with status epilepticus were compared and the Sodium valproate was more effective than phenytoin (11). Moreover, in a study to determine the efficacy the Sodium valproate versus diazepam in children with status epilepticus, 40 children were studied. Seizure control occurred in 80% of patients' in-group receiving Sodium valproate and in 85% in-group receiving diazepam (12). 

The efficacy of Sodium valproate and phenytoin as the first-line treatment in status epilepticus were compared and showed that there was no significant difference between two groups in terms of seizure control, length of stay in hospital, mortality and serious cardiovascular complications (13).

Considering the importance of these cases, including seizure control and prevention of complications in children, the applicability of the Sodium valproate in the treatment of diseases associated with neurologic disorders and also, with attention to side effects of phenobarbital and conflicting results in reviewed studies. 

The current study was conducted to compare the effect of phenobarbital versus Sodium valproate in management of children with status epilepticus referred to Emergency Ward in Ali-Ebne Abitaleb Hospital in Zahedan, eastern Iran in 2013.

## Materials & Methods

This randomized single-blind clinical trial study registered with the code number IRCT2015051722300N1 was conducted in Emergency Ward in *Ali-Ebne Abitaleb Hospital *in Zahedan, eastern Iran in 2013. The study population was the all of the children with seizure not responded to diazepam within 5 min referred to Emergency Ward. Since the similar study was not found, the pilot study was conducted for calculation the sample size. Based on the results of pilot study with 95% confidence interval and the power of 0.9, sample size was determined as 39 persons in each group. Forty patients were studied in each group. 

Patients were selected conveniently and based on inclusion criteria. The most important inclusion criteria were as follows: children aged 6 months to 10 years with seizure lasts more than 5 min with no response to initial treatment to diazepam within 5 min (despite receiving one dose of diazepam 0.2 mg/kg). The second seizure after birth, lack of following conditions: history of serious reactions to Sodium valproate, history of uncontrolled bleeding, thrombocytopenia, active liver disease, heart rhythm disturbances, orthostatic hypotension, syncope, history of oral or injectable anticonvulsants, disease that prevents the use of valproate in children such as asthma, chronic liver disease, infection of the central nervous system and the lack of cryptogenic and symptomatic epilepsy. The exclusion criteria of the study included not receiving the full dose of medication for any reason.

The patients were randomly allocated into two phenobarbital and Sodium valproate groups through *permuted blocks*. Arranging the blocks were randomly and through using random numbers table and the patient entered into two groups based on the blocks. 

In intervention group (Sodium valproate), patients were given drug with dose of 20 mg/kg through IV infusion. If their seizure was controlled during 20 min, this was considered as a positive response to drug. The Sodium valproate purchased was constructed of Gerot Lannach Company in Austria. 

In control group, phenobarbital was given at dose of 20 mg/kg through IV infusion. If their seizure was controlled during 20 min, this was considered as a positive response to drug. Phenobarbital was from Chemi Darou Industrial Company.

Data collection tools were the demographic information form including (age, gender, history of previous seizure and type of seizure). Besides, the recovery time after receiving the drug was recorded in information forms in both groups.

Data were analyzed through using SPSS ver. 21 (Chicago, IL, USA), descriptive statistics (mean, SD and frequency), Chi-square test for comparing the qualitative variables in two groups, independent sample *t*-test for comparing the quantitative variables in two groups and *Pearson correlation coefficient. In all tests a significance level of 0.05 was considered.*

**Ethical Considerations**

The current study was approved in 2013 by the Research Ethics Committee of the univeristy. All patients’ relatives were provided with standardized information about the procedure. Informed consent was sought from patients’ relatives and they were guaranteed about the refuse of participation of their patients in the study. All codes of ethics in human research were respected.

## Results

Forty patients hospitalized with seizure were studied in intervention group (treatment with Sodium valproate) and 40 patients in control group (treatment with Phenobarbital). The mean age of patients in intervention group was 4.154.4 and in control group was 4.624.96 yr of no significant difference (*P*=0.652).

Moreover, in terms of gender, in intervention group 27 and in control group 25 patients were female. The chi-square test showed no significant difference (*P*=0.639).

The frequency distribution of seizures type showed that in both groups, the most common seizure was status epilepticus. Besides, two groups were similar in terms of type of seizure at the beginning of the study and chi-square test did not show significant difference between the two groups (Table 1).

In the phenobarbital group, the higher percent of patients had positive response to drug in compare with Sodium valproate group. Moreover, this difference was statistically significant between two groups in terms of frequency of positive response to treatment (Table 2). 

The mean response time in Sodium valproate was higher than the phenobarbital group. In fact, in the Sodium valproate group positive response to drug lasted longer time than phenobarbital group. However, the t-test showed no significant difference between two groups in terms of response time (*P*=0.06).

In addition, the mean response time to drug in both groups was assessed according to type of seizure. In phenobarbital group, response to drug in patients with SE lasted longer time than the patients with FC and this difference was significant (*P*=0.04). Unlike, in Sodium valproate group, response to drug in patients with FC lasted longer time than the patients with SE, but this difference was not significant (Table 3).

The relationship between age and response time to treatment revealed that with increasing the age of patients, response time to treatment also was increased and Pearson correlation test showed a significant direct relationship between the two variables (r=0.33, *P*=0.018). 

Comparison of relationship between response to treatment and gender of patients showed that in the intervention group among the 27 female children, 8 patients had the positive response and among 13 male children, 10 patients had positive response to treatment and chi-square test showed the significant difference (*P*=0.005). However, in the control group, among the 25 female children, 19 patients had the positive response and among 15 male children, 13 patients had positive response to treatment, but there was no significant difference (*P*=0.413).

Besides, comparison the mean and standard deviation of response time concerning the gender in both groups showed that this mean was more in female children than male children in intervention group were, but the *t*-test did not show significant difference. In control group, this time in female patients was more than the male patients and t-test showed no significant difference (Table 4).

## Discussion

The present research aimed to compare the effect of Phenobarbital versus Sodium valproate in management of children with status epilepticus referred to emergency ward. The patients in phenobarbital group had more positive response than the patients in Sodium valproate group. This means that the Sodium valproate was less effective than phenobarbital for control of seizure in our study sample.

Assessing the mean response time to drug according to seizure type in two groups showed that in phenobarbital group. This time in patients with FC was lower in compared with the patients with SE. In the other words, the patients with febrile convulsion had better response to phenobarbital in compared with patients with status epilepticus. 

**Table 1:** Comparing the type of seizure in two Sodium valproate and phenobarbital groups

Seizure type	FC (%)	SE (%)	Total (%)
Group			
**Sodium Valproate**	15 (37.5)	25 (62.5)	40 (100)
**Phenobarbital**	18 (45)	22 (55)	40 (100)
**Chi-square test**	*P*-value= 0.496 df=1 X^2^= 0.464		

**Table 2:** Comparing the frequency of positive response to drug in two groups

Response to drug	Sodium valproate (%)	Phenobarbital (%)
Group		
**Positive**	18 (45)	32 (80)
**Negative**	22 (55)	8 (20)
**Total**	40 (100)	40 (100)
**Chi-square test**	X^2^= 10.45 df= 1 *P*-value= 0.001	

**Table 3:** Comparing the mean response time to drug according to seizure type in two groups

Group	Response time	Number	Mean SD	Test result
	Seizure type			
Phenobarbital	FC SE	18 22	4.331.78 5.812.71	*t*=1.89 df= 38 *P-*value= 0.04
Sodium valporate	FC SE	15 25	5.932.46 4.242.89	*t*=-1.99 df= 38 *P*-value= 0.06

**Table 4:** Comparing the mean and SD of response time in male and female patients in two groups

Response time		MeanSD	*t*-test result
Group			
Sodium valproate	Female male	6.252.31 6.13.57	*t*=0.102 df=16 *P-*value= 0.92
Phenobarbital	Female male	4.722.19 4.692.72	*t*= 0.034 df= 29 *P-*value =0.973

Unlike, in Sodium valproate group, patients with status epilepticus had more rapid response to drug in compared with patients with febrile convulsion. In fact, patients with status epilepticus had better response to Sodium valproate in compared with patients with febrile convulsion. These different responses to drug can be attributed to type of seizure in patients. The effectiveness of Sodium valproate and phenytoin was compared; the mean of response time to drug in Sodium valproate was less than in phenytoin. In this study, the patients were suffering from status epilepticus (13) corroborated the results of current study. 

The main goal of treating is to completely control seizures during 20 minutes after starting the anticonvulsant infusion. A high percent of patients with status epilepticus had a better response to Sodium valproate in compare to phenobarbital (14) that was similar to results of current study. In our study, the patients with SE had a better and more rapid response to Sodium valproate in comparison with patients with FC. In patients with status epilepticus, Sodium valproate can be more effective than phenobarbital. 

The effectiveness of Sodium valproate and phenytoin was compared in patients with status epilepticus aged 13 to 60 yr old. The response to treatment was 25% and 79% in phenytoin and Sodium valproate respectively (11) corroborated the results of current study.

Furthermore, aim of compare the effectiveness of Sodium valproate and phenytoin in patients with seizure aged 15 to 50, seizure was controlled in 66% of patients receiving Sodium valproate and in 68% phenytoin. The results of two recent studies indicated the effectiveness of Sodium valproate as an anticonvulsant drug for treatment of status epilepticus (15).

The effectiveness of Sodium valproate and phenytoin was compared as second-line treatment of seizure control, the positive response in Sodium valproate was 88% and in phenytoin group was 84% (16). The effectiveness of Sodium valproate was determined on 13 patients with status epilepticus aged 4-12 yr old, 63.3% of patients had positive response to drug (17). Intravenous Sodium valproate can be used as the first choice in the treatment of SE and acute repetitive seizures in children (18).

There was a significant direct relationship between the age and response time in all of the patients. In other words, with increasing the patient’s age, the response time to drug was increasing that is similar to results that the children with higher mean age had more rapid positive response to drug in compared with younger children (17).

Generally, by comparing the results of various studies with current study, both Sodium valproate and phenobarbital are effective drugs in second-line treatment in patients with seizures especially with regard to the type of seizure. The Sodium valproate had more effectiveness, the type of seizure was status epilepticus, but the phenobarbital had been more effective, the type of seizure was generalized and partial. In our study, valproate Sodium was more effective in patients with status epilepticus. 

**In conclusion, **the phenobarbital in comparison with Sodium valproate is a suitable and effective drug for controlling of seizure in children. 
